# IL-17A regulates *Eimeria tenella* schizont maturation and migration in avian coccidiosis

**DOI:** 10.1186/1297-9716-45-25

**Published:** 2014-02-26

**Authors:** Emilio del Cacho, Margarita Gallego, Hyun Soon Lillehoj, Joaquín Quílez, Erik P Lillehoj, Ana Ramo, Caridad Sánchez-Acedo

**Affiliations:** 1 Department of Animal Pathology, Faculty of Veterinary Sciences, University of Zaragoza, Zaragoza, Spain; 2 Animal Biosciences and Biotechnology Laboratory, Beltsville Agricultural Research Center, Agricultural Research Service, U.S. Department of Agriculture, Beltsville, MD 20705, USA; 3 Department of Pediatrics, University of Maryland School of Medicine, Baltimore, MD 21201, USA

## Abstract

Although IL17A is associated with the immunological control of various infectious diseases, its role in host response to *Eimeria* infections is not well understood. In an effort to better dissect the role of IL17A in host-pathogen interactions in avian coccidiosis, a neutralizing antibody (Ab) to chicken IL17A was used to counteract IL17A bioactivity in vivo. Chickens infected with *Eimeria tenella* and treated intravenously with IL17A Ab, exhibited reduced intracellular schizont and merozoite development, diminished lesion score, compared with untreated controls. Immunohistological evaluation of cecal lesions in the parasitized tissues indicated reduced migration and maturation of second-generation schizonts and reduced lesions in lamina propria and submucosa. In contrast, untreated and infected chickens had epithelial cells harboring second-generation schizonts, which extend into the submucosa through muscularis mucosa disruptions, maturing into second generation merozoites. Furthermore, IL17A Ab treatment was associated with increased parameters of Th1 immunity (IL2- and IFNγ- producing cells), reduced levels of reactive oxygen species (ROS), and diminished levels of serum matrix metalloproteinase-9 (MMP-9). Finally, schizonts from untreated and infected chickens expressed S100, Wiskott-Aldrich syndrome protein family member 3 (WASF3), and heat shock protein-70 (HSP70) proteins as merozoites matured, whereas the expression of these proteins was absent in IL17A Ab-treated chickens. These results provide the first evidence that the administration of an IL17A neutralizing Ab to *E. tenella*-infected chickens inhibits the migration of parasitized epithelial cells, markedly reduces the production of ROS and MMP-9, and decreases cecal lesions, suggesting that IL17A might be a potential therapeutic target for coccidiosis control.

## Introduction

The coccidial parasite, *Eimeria tenella*, is a significant cause of intestinal disease in chickens and hens worldwide*. Eimeria tenella* is an intracellular obligate protozoan parasite having a complex life cycle of seven days, during which it undergoes intracellular development and proliferates through characteristic intracellular stages confined to the cecal epithelium. *Eimeria tenella* initially invades the epithelial cells lining in the lumen of the crypts of Lieberkhün. The ensuing life cycle of the parasite involves detachment of the parasitized cells from the epithelial layer and their migration to the underlying connective tissue of the lamina propria (LP), where maturation to schizonts occurs [1]. Early development of schizonts is accompanied by considerable hypertrophy and the modification of the host cell, which survives in the LP as long as the schizont matures [1]. Schizont maturation takes place as the host cells harboring the parasite migrates through the LP deep into the muscularis mucosa (MM), the boundary between the LP and the submucosa.

The ability of parasitized epithelial cells to detach from the epithelium lining of the crypts, migrate through the basal membrane underneath the epithelium, and invade the LP relies on a complex cascade of molecular events involving a finely tuned interplay between parasite and host cells. Among the factors playing a role in this response, interleukin-17A (IL17A), a proinflammatory cytokine secreted by activated T helper cells (Th17 type), plays a critical role in host defense against pathogens [2-4]. IL17A transcripts were shown to be highly up-regulated during avian coccidiosis [5,6], and IL17A protein plays a proinflammatory role during noncoccidial protozoan infections [7-9]. Moreover, IL17A induces the production of reactive oxygen species (ROS) [10], and activates the epithelial cell contractile machinery through cytoskeleton rearrangements [11] and disruption of intercellular tight junctions [12,13]. It is precisely these properties of IL17A that led us to study its role on *E. tenella* schizonts maturation and intracellular stage progression in cecal coccidiosis. To evaluate the effects of IL17A on facilitating the detachment of the parasitized cells from the epithelial layer and promoting their subsequent migration through the LP, a IL17A neutralizing Ab [14] was administered to chickens infected with *E. tenella*. To further confirm the influence of IL17A on intracellular parasite development and the subsequent parasite-specific immune response, we also evaluated Th1- and Th2-cytokine responses. In addition, the effect of neutralizing local IL17A production on ROS generation in cecal cells was measured to evaluate the role of parasite-induced oxidative stress. Finally, we studied the expression of proteins having a role in cell motility and cytoskeleton rearrangement. These motility-associated proteins included Wiskott-Aldrich syndrome protein family member 3 (WASF3), which is implicated in actin polymerization and cell movement [15,16], heat shock protein-70 (HSP70), a chaperone involved in the folding of newly synthesized proteins and which directly influences WASF3 stability [15], and S100, which belongs to the super-family of acid Ca^2+^-binding proteins of the EF-hand type which regulates microtubule assembly/disassembly [17]. Moreover, serum MMP-9 levels were measured to evaluate the effect of the IL17A Ab on cell migration through the extracellular matrix. In this report, we provide first evidence that treatment of *E. tenella*-infected chickens with IL17A neutralizing Ab reduced the motility of schizont-harboring epithelial cells and decreased their migration to deep areas in the LP. These results demonstrate a role for IL17A in the life cycle of *E. tenella*, specifically on schizont maturation and migration.

## Materials and methods

### Animals

White Leghorn chickens were hatched and reared under *Eimeria*-free conditions with access to feed and water provided *ad libitum*. All experiments were performed in accordance with the guidelines approved by the University of Zaragoza Institutional Animal Care and Use Committee.

### Parasite

A strain of *E. tenella* originally obtained from Merck, Sharp and Dome (Madrid, Spain) was used. Oocysts were propagated, isolated, and sporulated using standard procedures [18]. Chickens were infected with sporulated oocysts that had been stored for less than 4 weeks by oral inoculation into the crop [19].

### Experimental design

One-day old chickens (*n* = 24) were randomly divided into three groups of eight chickens each with equal mean body weights. At 15 days of age, chickens in groups I and II were infected orally into the crop with 1.0 × 10^4^*E. tenella* sporulated oocysts per bird. At 3, 4, and 5 days post-infection (dpi), chickens in group I were intravenously injected into the radial vein with 3 μg per bird of IL17A Ab [14] and chickens in group II were intravenously injected with sterile PBS. Chickens in group III served as uninfected noninjected controls and chickens in group II served as noninjected infected controls. At day 5 post-infection (pi), three animals from each group were euthanatized and ceca samples were removed to visualize schizont lesions by both the light and electron microscopy, and blood was collected to quantify IL17A levels in serum. At day 6 pi, five animals from each group were euthanatized and ceca, cecal tonsil (CT), spleen, and Peyer’s patch (PP) samples were removed to visualize histopathological ceca lesions, quantify antigen-specific cytokine-secreting cells (IL2, IL4, IL10, and interferon (IFNγ), and measure ROS. In addition, at this time point, blood was collected for quantification of IL17A and MMPs. Ceca lesion scores were determined on a graded scale from 0 (none) to 4 (high) in a blinded fashion by two independent observers as described [20]. The complete experimental procedure was repeated three times.

### Quantification of IL17A in serum

Sera for IL17A measurement were collected at days 5 and 6 pi from chickens given a single oral dose of 1.0 × 10^4^ sporulated oocysts of *E. tenella*. Ninety-six-well flat-bottom microtiter plates were coated with 100 μL of 20 μg/mL of chicken IL17A Ab [14] in 0.1 M carbonate buffer, pH 9.6 for 18 h at 48 °C, and washed three times with phosphate buffered saline containing 0.05% Tween-20, pH 7.2 (PBS-T). Each well was blocked with 200 μL of PBS containing 2% (w/v) bovine serum albumin (BSA) for 1 h at room temperature, and washed three times with PBS-T. Sera (100 μL) were added, the plates incubated for 2 h at room temperature, washed three times, 100 μL of horseradish peroxidase (HRP)-conjugated goat anti-mouse IgG (H + L; Sigma, St. Louis, MO, USA) in PBS-0.1% BSA added, and incubated for 1 h at room temperature. The plates were washed three times, 100 μL of 0.01% (w/v) 3,3′,5,5′-tetramethylbenzidine dihydrochloride (TMB; Sigma) in 0.05 M phosphate-citrate buffer, pH 5.0, added for 15 min, the reaction stopped with 50 μL of 2 M H_2_SO_4_, and the absorbency at 450 nm read by an automated microtiter plate reader (Bio-Rad, Richmond, CA, USA).

### Immunohistochemistry

#### Double staining immunohistochemistry

Ceca samples were frozen in liquid nitrogen. Cryostat sections were made and blocked with normal horse serum for 10 min, rinsed in PBS, pH 7.2, and endogenous peroxidase was inactivated with 1.7% H_2_O_2_ in ethanol for 30 min. Sections were incubated with fibronectin rabbit Ab (Sigma) (1:100 dilution) at room temperature for 90 min. After washing with PBS, pH7.2, the slides were incubated with biotinylated goat anti-rabbit IgG Ab (Vector, Burlingame, CA, USA) for 30 min. The avidin-biotin peroxidase complex (ABC) (Vector) was applied for 45 min and the peroxidase reaction was developed with 0.05% of 3-amino-9-ethylcarbazole substrate in 0.05 M Tris–HCl, pH 7.6, containing 0.01% H_2_O_2_ for 10 min. Sections were rinsed in PBS, pH 7.2, and incubated with mouse caveolin-1 monoclonal Ab (Santa Cruz Biotechnology, CA, USA) (1:100 dilution) at room temperature for 90 min. After washing with PBS, the slides were incubated with biotinylated goat anti-mouse IgG Ab (Vector) for 30 min. The ABC (Vector) was applied for 45 min, and the binding sites of the primary Ab were visualized with 0.2 mg/mL of 3,3′-diaminobenzidine (DAB) in 0.05 M Tris–HCl, pH 7.6, containing 0.005% H_2_O_2_ for five min. As negative controls, sections of all samples were incubated with normal serum instead of the primary Ab, with the remaining procedure being the same. These negative control sections were devoid of positive-staining cells.

#### Single staining immunohistochemistry

Cryostat sections were rinsed in PBS, pH 7.2, and endogenous peroxidase was inactivated with 1.7% H_2_O_2_ in ethanol for 30 min. The slides were blocked with undiluted normal pig serum at room temperature for 30 min and incubated with primary Ab (1:10 dilution) at 4 °C for 18 h. As primary antibodies we used S100 mouse monoclonal Ab (Sigma), HSP70 mouse monoclonal Ab (Sigma) or WASF3 rabbit polyclonal Ab (Antibodies-Online, Atlanta, GA). After washing with PBS, pH7.2, the slides were incubated with biotinylated goat anti-mouse or goat anti-rabbit IgG Abs (Vector) for 30 min. The ABC was applied for 45 min and the peroxidase reaction was developed with DAB as above.

### ROS measurement

Measurement of hydroxyl, peroxyl and other ROS activities was performed using 2′,7′-dichlorofluorescein diacetate (DCFDA) with the ROS assay kit (Abcam, Cambridge, MA, USA). Cecal samples were harvested at day 6 post-immunization, pressed through 250 μm mesh screens, and resuspended and washed in GKN buffer (2.0 mg/mL glucose, 0.40 mg/mL KCl, 8.0 mg/mL NaCl, 3.56 mg/mL Na_2_HPO_4_, 0.78 mg/mL NaH_2_PO_4_, pH 7.4) containing 5.0 mM EDTA. Single-cell suspensions were obtained by filtration through a 70 μm pore size cell strainer (BD Falcon, Franklin Lakes, NJ, USA) and were purified from dead cells, erythrocytes, and epithelial cells by Percoll density gradient centrifugation [21]. Approximately 2.5 × 10^4^ cells were incubated in 20 μM DCFDA for 45 min at 37 °C according to the manufacturer′s instructions. Incubated cells were then washed with PBS, transferred to microplates, and read on a microplate fluorometer (λ_ex =_ 485 nm, λ_em_ = 535 nm).

### Electron microscopy

Cecal samples were fixed in glutaraldehyde for 90 min, washed in Millonig buffer, and fixed for 30 min in 1% osmium tetroxide [22]. After a final washing in buffer and dehydration in ethanol, the fixed tissue was cleared in propylene oxide and embedded in Epon-Araldite (1:1) (Sigma). Semi-thin (1 μm) and ultrathin (40–60 nm) sections were made and stained with toluidine blue, uranyl acetate and lead citrate.

### Quantification of MMP-9

Sera for MMP-9 measurement were collected at day 6 pi from uninfected and infected chickens given a single oral dose of 1.0 × 10^4^ sporulated oocysts of *E. tenella*. Ninety-six well flat-bottom microtiter plates were coated with 100 μL of 20 μg/mL of MMP-9 polyclonal Ab (MyBioSource, San Diego, CA, USA) in 0.1 M carbonate buffer, pH 9.6 for 18 h at 48 °C, and washed three times with PBS-T. Each well was blocked with 200 μL of PBS-BSA) for 1 h at room temperature, and washed three times with PBS-T. Sera (100 μL) were added, the plates incubated for 2 h at room temperature, washed three times, 100 μL of HRP-conjugated goat anti-rabbit IgG Ab (H + L; Sigma) in PBS-0.1% BSA added, and incubated for 1 h at room temperature. The plates were washed three times and developed with TMB as above.

### Quantification of *E. tenella* antigen-specific cytokine-secreting cells

CT, spleen, and PP samples were separately harvested at day 6 post-immunization, pressed through 250 μm mesh screens, resuspended and washed in GKN buffer containing 5.0 mM EDTA, and single cell suspensions were purified by Percoll gradient centrifugation as above. For quantification of cytokine-producing cells, 1.0 × 10^5^ cells were cultured for three days in the presence or absence of *E. tenella* antigens (100 μg/mL) [22] in 0.2 mL of RPMI 1640 medium. The cells were washed and added to microplates that had previously been coated overnight at 4 °C with chicken IL2, IL4, IL10, or IFNγ Abs, blocked at room temperature for 2 h with PBS containing 10 μg/mL of BSA and 0.05% Tween 20 (PBS/BSA/T), and washed three times with PBS. Cytokine enzyme-linked immunosorbent spot (ELISPOT) assays were performed as described [22].

### Statistical analysis

Statistical analysis was performed using SAS System for Windows V.9.2. All data were expressed as mean ± standard deviation (SD) values. Duncan′s multiple-range test was used to evaluate the differences between treatment groups. Differences between mean values were considered statistically significant at *P* < 0.05.

## Results

### IL17A neutralizing Ab reduces *E. tenella*-induced serum IL17A

**Figure 1:**
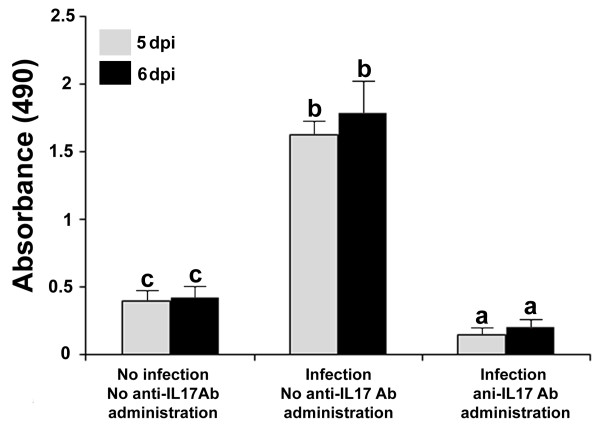
**IL17A neutralizing Ab reduces *E. tenella*-induced serum IL17A.** Blood samples were collected at 5 days (white bars) and 6 days (black bars) pi from untreated and uninfected, infected and untreated, and infected and IL17A Ab-treated chickens. Each bar represents the mean ± SD value from three independent experiments. Within each graph, bars with different letters are significantly different (*P* < 0.05) according to Duncan’s multiple-range test.

Serum IL17A levels in *E. tenella*-infected chickens at days 5 and 6 pi were increased compared with uninfected controls (Figure 1). However, IL17A levels were significantly decreased in infected chickens that were given 3 μg per bird of IL17A neutralizing Ab at 3, 4, and 5 dpi, compared with infected nontreated controls.

### Effect of IL17A Ab on schizont migration through the cecal wall

**Figure 2:**
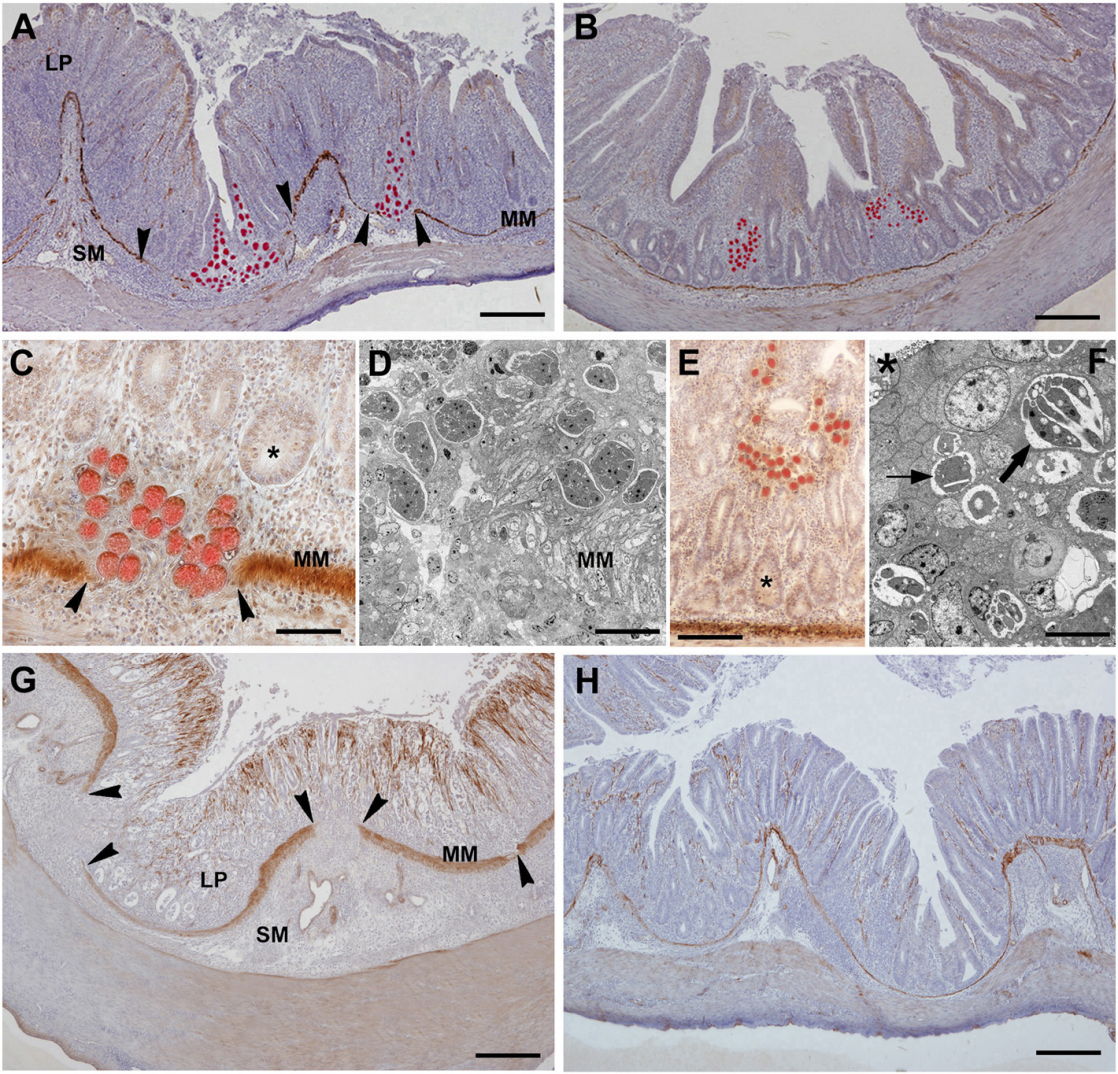
**Effect of IL17A Ab on *E. tenella *schizont migration.** Cecal tissue sections from infected chickens were taken at 5 days **(A-F)** and 6 days **(G,H)** pi. Schizonts are observed as round and red structures expressing fibronectin in chickens untreated **(A,C,D,G)** or IL17A Ab-treated **(B,E,F,H)**. The muscularis mucosa (MM) is visualized as a thin and brown caveolin positive line beneath the base of the crypts. **A**. Migration of schizonts from the crypt epithelium to the submucosa (SM) through MM disruptions (arrowheads). Note the lamina propria (LP) diffusely infiltrated by numerous immune system cells which extend into the SM. **B**. Schizont groups which do not reach the SM. Note the cell infiltrates confined to the LP and the continuity of the MM. **C**. Schizonts, which locate deep in the LP, extend beyond the MM. Lumen of crypt fundi (*). **D**. Electron micrograph showing the lower portion of the LP where immature schizonts are recognizable. **E**. Schizonts observed in the upper portion of the LP. Lumen of crypt fundi (*). **F**. Electron micrograph showing schizonts recognizable as mature (thick arrow) and immature stages (thin arrow). Lumen of a crypt (*) **G**. Prominent cell infiltrates in the LP and SM. Arrowheads: MM disruptions. H. Light cell infiltrates located in the LP surrounding the crypts. Note inconspicuous infiltrates in the SM and the continuous MM. Bars indicate: 300 μm in **A** and **B**; 130 μm in **C**; 50 μm in **D**; 90 μm in **E**; 10 μm in **F**; 230 μm in **G**; 270 μm in **H**.

At day 5 pi, when large numbers of schizonts are present in the cecal mucosa, fibronectin-expressing schizonts were identified as they migrated from the epithelial lining of the crypts of Lieberkühn through the cecal wall (Figures 2A and 2B). In infected chickens that were not given the IL17A Ab, schizonts were seen to reach the intestinal MM, the deepest portion of the mucosa (Figures 2A and 2C). In places, the MM was disrupted (Figures 2A and 2C) and the schizonts extended into the submucosa (SM) (Figures 2A and 2D). Schizonts were clearly observed on both sides of the MM, the mucosa, and SM. These migrating schizonts were mainly immature (Figure 2D). Notably, the LP and SM contained numerous round cells of the immune system, which were observed as prominent dense infiltrates (Figures 2A and 2C). However, in infected chickens treated with IL17A Ab, the LP between the crypts and within the core of each villi contained less cell infiltration (Figures 2B and 2E). In addition, epithelial cells harboring schizonts and migrating through the LP did not extend to the SM in IL17A Ab treated chickens. The MM was observed as a continuous line separating the mucosa from the submucosa (Figures 2B and 2E). Thus, in infected chickens given IL17A Ab, schizonts were confined within the LP near the epithelium lining the cecal lumen (Figures 2B, 2E, and 2F). These schizonts were both immature and mature, the later being recognized by their development into merozoites (Figure 2F).

At day 6 pi, when merozoites were formed and schizont numbers dramatically decreased, the cecal wall was devoid of immunoreactivity for fibronectin (Figures 2G and 2H). In untreated infected chickens (Figure 2G), large numbers of inflammatory cells were observed in both the LP and SM, and the cecal wall was thickened. In addition, frequent disruptions in the MM were observed (Figure 2G). However, in infected chickens that were given IL17A Ab, the round cell infiltrates were fairly inconspicuous within both the LP and SM, and the MM was seen as a continuous line devoid of disruptions (Figure 2H).

### Effect of IL17A Ab on expression of proteins involved in cell motility

**Figure 3:**
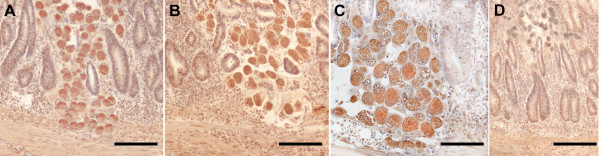
**Effect of IL17A Ab on the expression of S100 (A), HSP70 (B), and WASF3 (C) proteins by *E. tenella *schizonts.** Light micrographs showing cecal wall sections taken 5 dpi from untreated chickens **(A,B,C)**. Samples taken from IL17A Ab-treated chickens lacked of positivity for those three motility-related proteins as exemplified in **D**, where schizonts are devoid of positivity for S100 protein. Note the expression of those proteins versus the lack of positivity in IL17A Ab-treated chickens **(D)**. Bars indicate: 200 μm in **A**, **B** and **D**; 100 μm in **C**.

In the cecal wall of untreated infected chickens, both mature and immature schizonts, which were observed migrating through the LP, expressed S100, HSP70 and WASF3, proteins involved in cell motility (Figure 3A-C). However, no detectable immunostaining was observed in the tissue samples of IL17A Ab treated birds incubated with anti-WASF3, -HSP70 or -S-100 (Figure 3D).

### Effect of IL17A Ab on morphological quantification of schizonts

**Figure 4:**
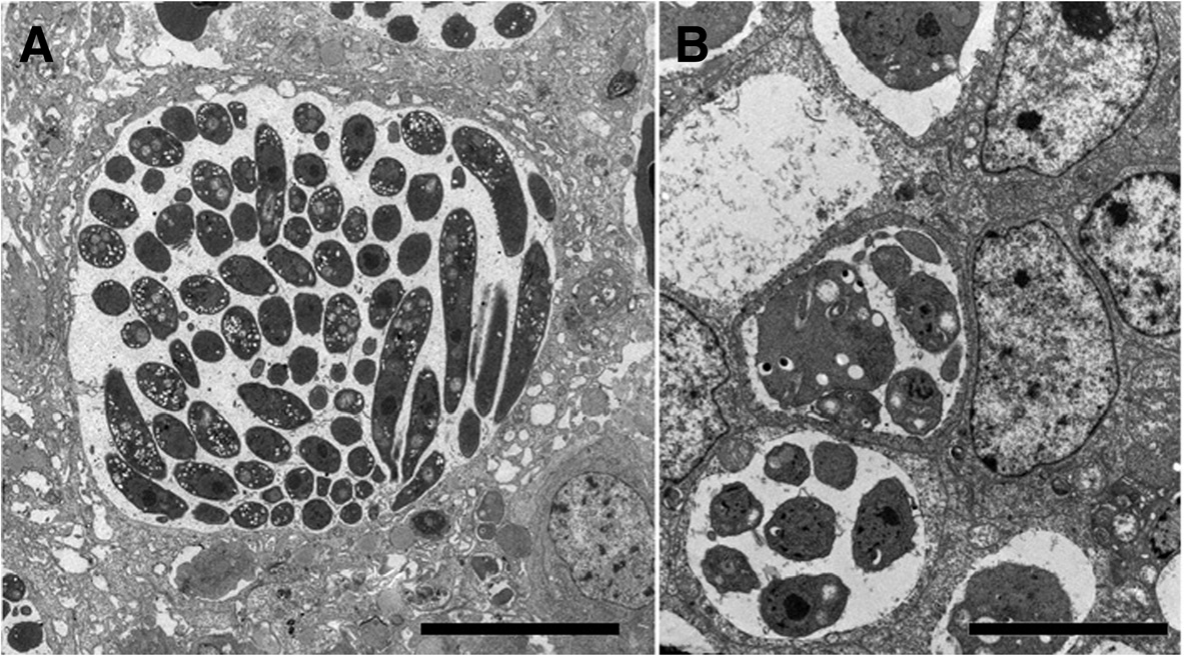
**Electron microscopy of hypertrophied and modified host cells harboring mature *E. tenella *schizonts.** Samples taken at 5 dpi from chickens that were not **(A)** or were given **(B)** IL17A Ab. Note the differences in diameter and numbers of merozoites. Bars indicate: 15 μm in **A**; 7 μm in **B**.

Schizonts observed in the cecal wall from untreated infected chickens were 32 ± 3.7 μm in diameter and contained 103 ± 9.1 merozoites per schizont (Figure 4A). However, schizonts seen in the cecal wall from infected chickens that were given the IL17A Ab had diameters of 7.5 ± 1.8 μm and contained 9.4 ± 2.8 merozoites per schizont (Figure 4B).

### Effect of IL17A Ab on ROS production by cecal cells

**Figure 5:**
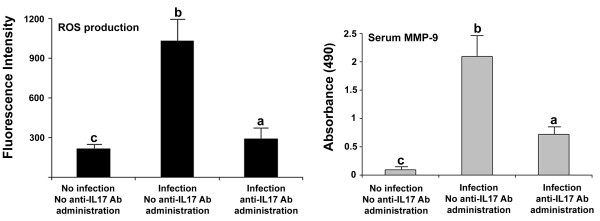
**ROS production and serum MMP-9 levels measured by fluorometry and ELISA, respectively.** Samples were taken at 6 dpi. Each bar represents the mean ± SD value from three independent experiments. Within each graph, bars with different letters are significantly different (*P* < 0.05) according to Duncan’s multiple-range test.

Increased ROS production was seen at day 6 pi in ceca-derived cells of untreated chickens, compared with untreated and uninfected chickens (Figure 5). Significant decreased ROS production was observed in ceca-derived cells from infected chickens that were given IL17A Ab, compared with infected birds that were not given Ab.

### Effect of IL17A Ab on serum MMP-9 levels

Increased MMP-9 levels were present at day 6 pi in sera of untreated and infected chickens, compared with untreated and uninfected chickens (Figure 5). Significantly decreased serum MMP-9 levels were observed in infected chickens that were given IL17A Ab, compared with infected and untreated birds.

### Effect of IL17A Ab on *E. tenella*-stimulated cytokine-producing cells

**Figure 6:**
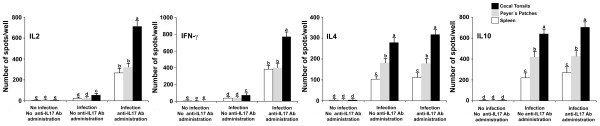
***E. tenella *antigen-specific T**_**h**_**1 and T**_**h**_**2 cytokine-secreting cells quantified by ELISPOT assay.** Samples were taken from the spleen, cecal tonsils, and Peyer’s patches at 6 dpi. Each bar represents the mean ± SD value from three independent experiments. Within each graph, bars with different letters are significantly different (*P* < 0.05) according to Duncan’s multiple-range test.

CT, PP, and spleen cells of infected chickens that were injected with IL17A Ab had significantly increased numbers of IL2- and IFNγ-producing cells, compared both with the uninfected/untreated and infected/untreated controls (Figure 6). The numbers of Th1 cytokine-producing cells were greater in CT-derived cells than in PP- or spleen-derived cells. This differential profile of cell response correlates with the site of infection in the intestine by *E. tenella*, the cecum. Infected animals had significantly increased the numbers of IL4- and IL10- producing cells in all three types of tissues examined, compared with uninfected chickens. However, administration of IL17A Ab did not alter the numbers of IL4- and IL10-producing cells in the CTs, PPs, and spleen, compared with the infected controls that were not given IL17A Ab (Figure 6).

### Effect of IL17A Ab on in vivo parameters of protective immunity against *E. tenella* infection

Infected chickens that were given IL17A Ab had decreased intestinal lesion scores, compared with infected chickens that were not given IL17A Ab (Table 1).

**Table 1:** **Effect of anti-IL17A Ab on *E. tenella *** **infection.**

**Treatment group**	**Lesion score (scale of 0–4)**
No infection, no anti-IL17A Ab administration	0^a^
Infection, no anti-IL17A Ab administration	2.5 ± 0.49^b^
Infection, anti-IL17A Ab administration	1.1 ± 0.4^c^

Each value represents the mean ± SD of three independent antibody administration/infection trials. Within each parameter values with different superscripts are significantly different (*p* < 0.05) according to Duncan’s multiple range test. The statistical analysis detected significant differences among experimental groups. Birds infected and inoculated with anti-IL17A Ab showed significant lower lesion scores than infected and non-inoculated birds (*p* < 0.05).

## Discussion

IL17A exerts critical immunological control of the host response to various infectious pathogens [23,24]. Although IL17A is known to be involved in the host response to protozoal infections [7,9], and its production has been associated with the protective response to coccidial infections [25], the specific effect of IL17A on *E. tenella* infection has not been clearly defined. Indeed, IL17A production has been suggested to be deleterious in avian coccidiosis [26], indicating the necessity of further research to discern the precise role of IL17A in *Eimeria* infections. Herein, we provide evidence that IL17A is involved in the initiation and migratory response of epithelial cells harboring *E. tenella* second-generation schizonts during intracellular development. Furthermore, our results indicate that IL17A contributes to the maturation of *E. tenella* schizonts contributing to severe cecal lesions, as demonstrated by the finding that the in vivo treatment with an IL17A neutralizing Ab led to increased parameters of Th1 immunity and reduced ROS and MMP-9 levels.

It is well known that epithelial cells harboring second-generation schizonts migrate deep into the mucosa in order for the schizonts to mature and form second-generation merozoites during intracellular development. The present study provides the first evidence that the administration of an IL17A Ab inhibited the migration of parasitized cells and markedly reduced the production of ROS and MMP-9, which facilitate cell migration through the extracellular matrix [10,27]. ROS have been implicated in oxidative stress leading to activation of the cell contractile machinery, which in turn, is responsible for the loss and disorganization of tight junction proteins, responsible for intercellular adhesion of cecal epithelial cells [10]. Treatment of *E. tenella*-infected chickens with IL17A Ab reduced ROS production, suggesting that the reduction of the migration of parasitized epithelial cells in the crypts is due to the lack of interference with the intercellular adhesion. A second step for parasitized cells in their migration towards the LP is to traverse through the basement membrane, the structural attachment site for overlying epithelial cells and underlying extracellular matrix and connective tissue. MMPs constitute a family of Zn^2+^-dependent endopeptidases that regulate the degradation, turnover, and processing of proteins of the extracellular matrix including fibrillar and non-fibrillar collagens, fibronectin, laminin, and basement membrane or interstitial stromal glycoproteins [27,28]. Treatment of *E. tenella*-infected chickens with IL17A Ab reduced MMP-9 production, suggesting hindered movement of host cells harboring second-generation schizonts into the connective tissue, as evidenced by the increased numbers of host cells containing mature second-generation schizonts near the epithelium lining the crypts. Importantly, in untreated infected chickens, increased MMP-9 production was found compared with uninfected controls. These data, plus the finding that cells harboring second-generation schizonts reached the deepest portion of the cecal mucosa, accumulated close to the MM, and extended into the SM, led us to suggest that the integrity of both intracellular junctional complexes and the basement membrane are impaired as a consequence of the increase in ROS and MMP-9 production during *E. tenella* infection. Furthermore, increased MMP-9 production facilitated the movement of cells harboring second-generation schizonts through the connective tissue in the LP. These results suggest that during *E. tenella* infection, IL17A promotes the migration of parasitized epithelial cells by facilitating their separation from the epithelial layer and movement away from the epithelium into the LP.

There is general consensus that the administration of IL17A neutralizing Ab reduces the degree of tissue damage of the cecal wall caused by *Eimeria* infections [26,29]. However, there are controversial reports concerning the effect of IL17A neutralization on fecal oocyst shedding. In murine coccidiosis, Stange et al. [29] reported that neutralization of IL17A production increased *E. falciformis* oocyst output. In contrast, Zhang et al. [26] reported reduced *E. tenella* oocyst shedding after IL17A neutralization in avian coccidiosis. Our findings are in accordance with the studies by Zhang et al. [26] indicating that the neutralization of IL17A dramatically decreased the numbers of merozoites and reduced the severity of the cecal tissue damage in cecal coccidiosis. Furthermore, our study suggests that decreased merozoite multiplication and cecal tissue damage may be caused by the reduced motility of cells containing second-generation schizonts. The numbers of oocyst produced depend upon the numbers of merozoites formed during schizogony [30]. We found that schizonts from IL17A Ab-treated chickens were small, and that merozoite numbers were reduced, compared to untreated infected chickens. In addition, parasitized epithelial cells did not move deep into the LP and did not trespassed the MM in IL17A Ab-treated chickens. Thus, limited migration caused less adverse effects, such as decreased disruption of blood vessels, and reduced tissue destruction and cell infiltration. On the basis of the findings discussed above, in infected chickens treated with IL17A Ab, reduced merozoite formation was associated with the inhibition of migration of parasitized cells and the impairment of schizont maturation.

In *E. tenella* infections, the migration of epithelial cells harboring second-generation schizonts is required for schizont maturation. Whereas the host cells moves through the LP, schizonts reproduce asexually by multiple fission to form a number of infective merozoites. These escape from the host cell and invade new cells [31]. The present findings also showed that schizonts from infected chickens not given the neutralizing Ab expressed S100, WASF3, and HSP70 proteins. WASF3 coordinates the development of lamellipodia at the leading edges of cells, and its loss prevents cell motility [32]. S100 and WASF3 are involved in actin polymerization and in cell motility [15,33,34]. HSP70 is a chaperone protein associated with both cell motility [15] and parasite capability to adapt to the new environment in the host [35]. HSP70 and its associated proteins involved in cytoskeleton rearrangement have been reported as regulators of infectivity and pathogenicity of *E. tenella*[36,37]. In our study, schizonts from infected chickens treated with IL17A Ab did not express these proteins, suggesting that during *E. tenella* infection locally produced IL17A promotes the expression of S100, WASF3, and HSP70 as the merozoites reach their infective capability.

Our study, as well as previously reported findings [26,29], demonstrates that neutralization of IL17A increased the Th1 cytokines, IL2, IFNγ, and IL12. These results may be associated with the fact that Th17 cells are antagonistic of Th1 cells. Indeed, IL17A and IL22, a Th17 cell-associated proinflammatory cytokine, have been reported to inhibit the production of Th1 mediated cytokines [38]. Since the Th1 response is the most efficient host response against *Eimeria* infection, it is conceivable that neutralization of IL17A increases the efficacy of the Th1 response against *E. tenella*. Although Th17 cells have been reported to be the major effector T cells that provide immunity in the intestine [39] against many potential pathogens [40,41], the exact roles of Th17 cells in the intestine are incompletely understood. Studies by Zhang et al. [26] and Stange et al. [29], in addition to our current report, support the notion that IL17A has a role in *E. tenella* pathogenesis. In summary, the present study confirms and extends previous observations in avian coccidiosis that neutralization of IL17A reduced *E. tenella* multiplication and diminished tissue lesions on the basis of a herein demonstrated reduction of parasitized epithelial cell motility. Consequently, IL17A might be a potential therapeutic target for avian coccidiosis control.

## Abbreviations

LP: Lamina propria; MM: Muscularis mucosa; CT: Cecal tonsil; PP: Peyer’s patch; Ab: Antibody; IL17A: Interleukin 17A; ROS: Reactive oxygen species; MMP-9: Matrix metalloproteinase-9; WASF3: Wiskott-Aldrich syndrome protein family member 3; HSP70: Heat shock protein 70; HRP: Horseradish peroxidase; TMB: Tetramethylbenzidine dihydrochloride; ABC: Avidin-biotin peroxidase complex; DAB: Diaminobenzidine; DCFDA: Dichlorofluorescein diacetate; ELISPOT: Enzyme-linked immunosorbent spot; SD: Standard deviation.

## Competing interests

The authors declare that they have no competing interests.

## Authors’ contributions

HSL, EC, CSA conceived and designed the study. JQ, MG participated in its coordination. AR, JQ, EC immunized and challenged the chickens and performed their clinical examination. HSL, EPL developed anti-chicken IL17A, IL-4, IL-10, and IFN-γ antibodies and interpreted the immunological results. MG, EC, AR, JQ, CSA participated in the collection and processing of the samples, and carried out the histopathological and immunological analysis. JQ, AR, CSA performed the statistical analyses and interpreted the results. HSL, EPL, MG, wrote the manuscript; with inputs from all authors. All authors read and approved the manuscript.
